# Endophytic Bacterial and Fungal Communities of Spruce *Picea jezoensis* in the Russian Far East

**DOI:** 10.3390/plants14162534

**Published:** 2025-08-14

**Authors:** Nikolay N. Nityagovsky, Alexey A. Ananev, Andrey R. Suprun, Alina A. Dneprovskaya, Konstantin V. Kiselev, Olga A. Aleynova

**Affiliations:** 1 Laboratory of Biotechnology, Federal Scientific Center of the East Asia Terrestrial Biodiversity, Far Eastern Branch of the Russian Academy of Sciences, 690022 Vladivostok, Russia; ananev.all@yandex.ru (A.A.A.); dneprovskayaalina@yandex.ru (A.A.D.); kiselev@biosoil.ru (K.V.K.); 2 Institute of the World Ocean, Far Eastern Federal University, 690090 Vladivostok, Russia

**Keywords:** endophyte microbiome, coniferous trees, Yezo spruce, *16S*, *ITS1*, next-generation sequencing

## Abstract

A wide range of microorganisms, including endophytes, frequently interact with forest trees. The role of endophytes in industrial conifers has not been fully investigated. The Yezo spruce *Picea jezoensis* is widely used for logging in Russia and Japan. In this work, the endophytic communities of bacteria and fungi in healthy needles, branches, and fresh wood of *P. jezoensis* from Primorsky Territory were analyzed using metagenomic analysis. The results indicate that the diversity of endophytic communities in *P. jezoensis* is predominantly influenced by the specific tree parts (for both bacteria and fungi) and by different tree specimens (for fungi). The most abundant bacterial classes were Alphaproteobacteria, Gammaproteobacteria and Actinobacteria. Functional analysis of KEGG orthologs (KOs) in endophytic bacterial community using PICRUSt2 and the PLaBAse PGPT ontology revealed that 59.5% of the 8653 KOs were associated with plant growth-promoting traits (PGPTs), mainly, colonization, stress protection, bio-fertilization, bio-remediation, vitamin production, and competition. Metagenomic analysis identified a high abundance of the genera *Pseudomonas* and *Methylobacterium-Methylorubrum* in *P. jezoensis*, which are known for their potential growth-promoting activity in other coniferous species. The dominant fungal classes in *P. jezoensis* were Dothideomycetes, Sordariomycetes, and Eurotiomycetes. Notably, the genus *Penicillium* showed a pronounced increase in relative abundance within the fresh wood and needles of Yezo spruce, while *Aspergillus* displayed elevated abundance specifically in the fresh wood. It is known that some of these fungi exhibit antagonistic activity against phytopathogenic fungi. Thus, our study describes endophytic communities of the Yezo spruce and provides a basis for the production of biologicals with potential applications in forestry and agriculture.

## 1. Introduction

Endophytes are microorganisms, including fungi, bacteria, and viruses, that inhabit the tissues of healthy plants during the whole or part of their life cycle [1]. Endophytes can be found in different parts of plants without causing immediate disease symptoms [2]. A number of endophytic species have been shown to be mutualistic, which increases the resistance of the host plant to herbivores and pathogens [3]. While endophytes play a role in keeping plants healthy, plants provide nutrients to endophytes [4]. There are a number of ways in which endophytes can prevent plant disease, including outcompeting pathogens for resources, boosting plant defenses, releasing beneficial compounds, and enhancing plant development, often working in concert [5].

Knowledge about the distribution of endophytes within coniferous trees could be useful for developing tools related to the biological control of tree diseases and pests [6]. The role conifer endophytes play in the host tree vitality and growth is poorly understood. The composition of endophyte communities can vary among individual conifer trees of the same species [7]. Environmental factors such as moisture and increased temperature are thought to influence the structure and abundance of endophyte communities [8]. The genetic make-up of the host plant is also a factor influencing foliar endophytes [9]. Variation in plant factors, such as condensed tannins [10], and needle size, as well as the content of nutrients in spruce tissues, also play a role in influencing endophyte communities [11].

The spruce tree holds a significant position in the coniferous forests of the northern hemisphere. According to Farjon [12], it is a key player in the functioning of forest ecosystems. *Picea jezoensis* (Siebold et Zucc.) Carrière is a type of evergreen tree that can be found in China (specifically in Jilin), Russia (in Magadan, Kamchatka, the Kuril Islands, the Sakhalin Island, Primorsky and Khabarovsk Territory), Japan (in Hokkaido and Honshu), and North Korea. This tree typically grows in cold and damp temperate rainforests, ranging from sea level to 2700 m a.s.l. It thrives on podzolic soils with an annual precipitation of 1000 to 2500 mm. Recent data have shown that the bark of *P. jezoensis* is a rich source of valuable secondary metabolites—stilbenes (mainly *trans*-isorhapontin) [13]. Also, the stem, including bark, of *P. jezoensis* contains phenolic compounds, such as serratane-type triterpenoids, which exhibited significant anti-tumor-promoting effects on mouse skin carcinogenesis [14,15,16].

It is also known that some of the spruce endophytes are able to synthesize secondary metabolites that have antibacterial and fungicidal activity. For example, antifungal metabolites have been produced by endophytes of *Picea rubens* (red spruce) and *Picea mariana* (black spruce) from the Acadian forest [17,18]. In addition, some spruce endophytes are able to stimulate plant growth and to suppress the development of pathogens. So, the dark septate endophyte *Phialocephala sphaeroides* suppresses transcripts of conifer pathogens and promotes root growth in *Picea abies* (Norway spruce) [19]. Plant growth-promoting bacterial strains from lodgepole pine significantly increased the height and biomass of their natural host and a foreign host (hybrid white spruce) in a 540-day greenhouse trial [20]. Also, some spruce endophytes are capable of the synthesis of insecticidal metabolites. For instance, endophytes of white spruce *Picea glauca* were able to produce metabolites that are toxic to the spruce pest *Choristoneura fumiferana* (spruce budworm) [21].

At present, there is a lack of knowledge regarding the endophytes associated with *P. jezoensis*, a tree that is of great economic and environmental importance. Consequently, the objective of this research was to explore the diversity of endophytic bacteria and fungi found in different parts (needles, branches, fresh wood), different seasons (winter, summer), and two different tree specimens of Yezo spruce in the center of Primorsky Territory, Russian Far East.

## 2. Results

### 2.1. Illumina MiSeq Sequencing

A total of 1,990,546 bacterial and 1,647,521 fungal paired-end reads were obtained. After filtering, a total of 993,222 bacterial and 1,420,018 fungal sequences were produced from 24 spruce samples (Tables S1 and S2). The mean and median read numbers for bacterial data per sample were 41,384 and 41,867 sequences, respectively. As for the fungal data, the mean and median read numbers per sample were 59,167 and 56,980 sequences, respectively.

### 2.2. Diversity and Taxonomical Composition of Endophytic Communities of Bacteria in Various Tree Parts and Specimens of Picea jezoensis in Winter and Summer

**Figure 1:**
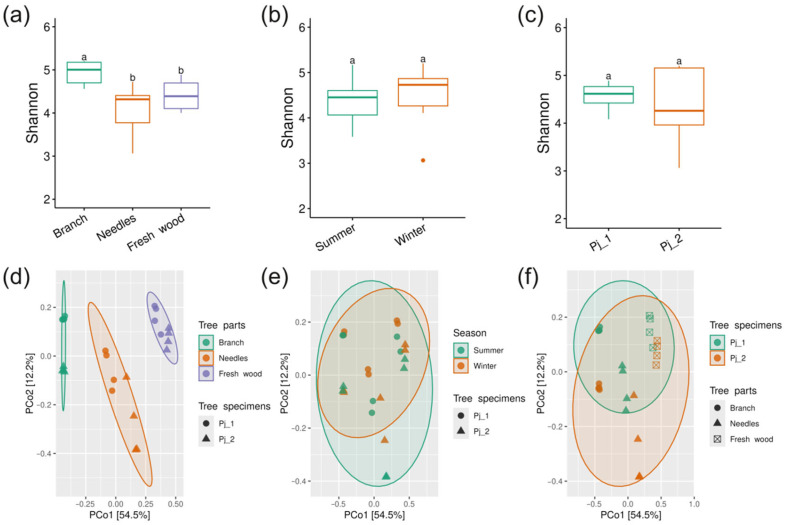
Shannon indexes of endophytic bacterial communities in different tree parts (**a**), seasons (**b**), and tree specimens (**c**). PCoA based on Bray–Curtis dissimilarity for endophytic bacterial communities in different tree parts (**d**), seasons (**e**), and tree specimens (**f**). Different lowercase letters indicate significant differences among Shannon indexes. Ellipses in PCoA plots show the 95% confidence intervals of a multivariate t-distribution.

Alpha diversity estimated by the Shannon index is significantly higher in branches than in needles and fresh wood (*p* < 0.05) (Figure 1a). The Shannon indices of bacterial communities in summer and winter do not differ statistically (Figure 1b). Similarly, no significant differences were observed between bacterial communities from tree specimens Pj_1 and Pj_2 (Figure 1c).

Principal Coordinate Analysis (PCoA) based on Bray–Curtis dissimilarity shows that the bacterial communities of branches, needles, and fresh wood exhibit significant separation, which is confirmed by the PERMANOVA analysis (R^2^ = 0.62, *p* < 0.01) (Figure 1d, Table S3). The analysis reveals overlapping clusters between bacterial communities from different tree specimens and seasons (Figure 1e,f). This is further supported by the lack of significant separation detected by PERMANOVA, suggesting similar bacterial community structures across different seasons and tree specimens of spruce (Table S3).

**Figure 2:**
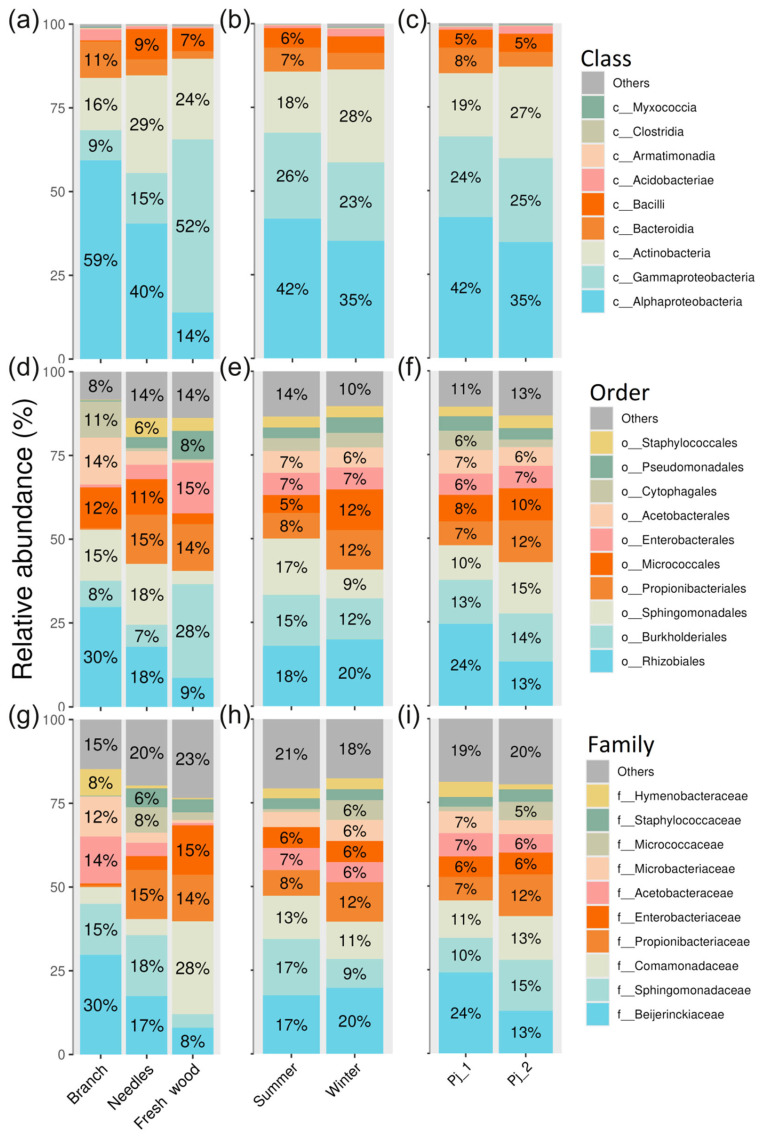
Composition of endophytic bacterial communities in *Picea jezoensis* at class (**a**–**c**), order (**d**–**f**), and family (**g**–**i**) levels in different tree parts (**a**,**d**,**g**), seasons (**b**,**e**,**h**), and tree specimens (**c**,**f**,**i**). The top 10 classes, orders, or families are shown. The remaining taxa are grouped into the other category.

The dominant bacterial classes in *P. jezoensis* belonged to Alphaproteobacteria, Gammaproteobacteria, and Actinobacteria (Figure 2a–c). The most abundant orders included Rhizobiales, Burkholderiales, Sphingomonadales, and Propionibacteriales (Figure 2d–f), while the dominant families were Beijerinckiaceae, Sphingomonadaceae, Comamonadaceae, and Propionibacteriaceae (Figure 2g–i).

**Figure 3:**
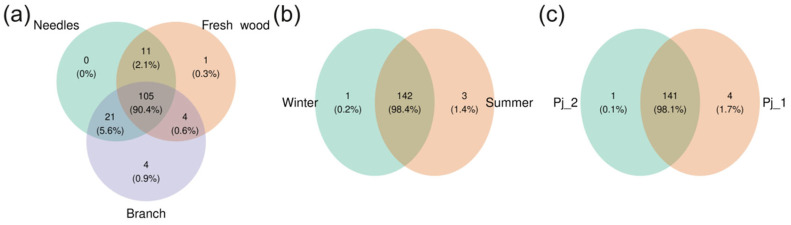
Venn diagrams illustrating shared and unique ASVs in bacterial communities of different tree parts (**a**), seasons (**b**), and tree specimens (**c**).

A total of 146 bacterial ASVs were found in the endophytic microbiome of *P. jezoensis* (Table S4). One hundred and five ASVs (90.4%) were common to all analyzed parts of the spruce, and one and four ASVs were unique to fresh wood and branches, respectively (Figure 3a, Table S5). One hundred forty-two endophytic bacterial ASVs (98.4%) were common in both seasons (Figure 3b, Table S6). One unique ASV was found in winter, and three in summer (Figure 3b, Table S6). For two different tree specimens, 141 endophytic bacterial ASVs (98.1%) were common (Figure 3c, Table S7). Four unique ASVs were found in Pj_1 plant, and one in Pj_2 plant (Figure 3c, Table S7).

**Figure 4:**
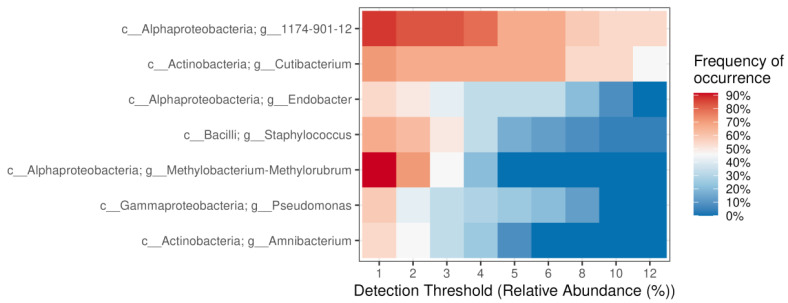
Core endophytic bacterial genera in *Picea jezoensis*. The “frequency of occurrence” represents the proportion of samples in which a genus was detected. Genera with relative abundance greater than 1% in at least 50% of the samples are displayed. “c__”—class, “g__”—genus.

Based on relative abundance > 1% in a minimum of 50% samples, the dominant genera of endophytic bacteria in *P. jezoensis* are *1174-901-12* (family Beijerinckiaceae), *Cutibacterium*, *Methylobacterium-Methylorubrum*, *Staphylococcus*, *Endobacter*, *Pseudomonas* and *Amnibacterium* (Figure 4). Less abundant bacterial genera detected in *P. jezoensis* include *Rothia*, *Bacillus*, *Terriglobus*, *Hymenobacter*, *Lactobacillus*, *Micrococcus*, *Burkholderia-Caballeronia-Paraburkholderia*, *Enhydrobacter*, *Corynebacterium*, *Escherichia-Shigella*, *Actinomycetospora*, *Sediminibacterium*, *Acidiphilium*, *Acinetobacter*, *37-13* (order Chitinophagales), *Spirosoma*, *Lawsonella*, *Acidibacter*, *Robbsia*, *Sphingomonas*, *Quadrisphaera*, *Sphingoaurantiacus*, *Massilia*, *Armatimonadales*, *Jatrophihabitans*, *Asinibacterium*, *Bradyrhizobium*, *Bryocella*, *Fenollaria*, and *Hydrotalea* (Table S4).

**Figure 5:**
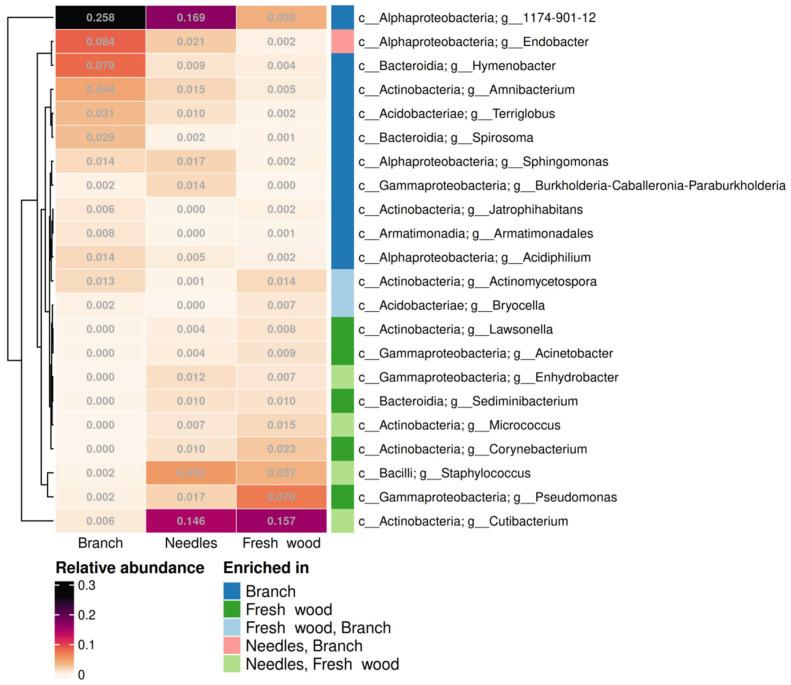
Endophytic bacterial genera that show a significant difference in their relative abundance among different tree parts of *Picea jezoensis*. “c__”—class, “g__”—genus. Distinct colors in the “Enriched in” column represent bacteria that are significantly enriched in different tree parts of *P. jezoensis* (*p* < 0.05).

According to the differential abundance test, 10 genera of endophytic bacteria were enriched in the branches, 5 in the fresh wood, 4 in the needles and fresh wood, 2 in the fresh wood and branches, and 1 in the needles and branches (*p* < 0.05) (Figure 5). Based on relative abundance, the enriched bacterial genera in branches are *1174-901-12* (family Beijerinckiaceae), *Hymenobacter*, *Endobacter*; in needles, *Cutibacterium* and *Staphylococcus*; and in fresh wood, *Cutibacterium* and *Pseudomonas* (*p* < 0.05) (Figure 5). *Escherichia-Shigella* were enriched in summer samples (*p* < 0.05) (Figure S1). No significant differences in bacterial genera abundance were detected in two spruce tree specimens.

### 2.3. Functional Analysis of Endophytic Bacterial Communities in Picea jezoensis

According to PICRUSt2 functional analysis, a total of 8653 KEGG orthologs (KOs) were predicted within the endophytic bacterial microbiome of *P. jezoensis*. To elucidate bacterial traits associated with plant growth promotion, the KOs were analyzed using the PLaBase PGPT ontology database. Out of 8653 KOs, 5150 (59.5%) were associated with plant growth-promoting traits (PGPTs).

**Figure 6:**
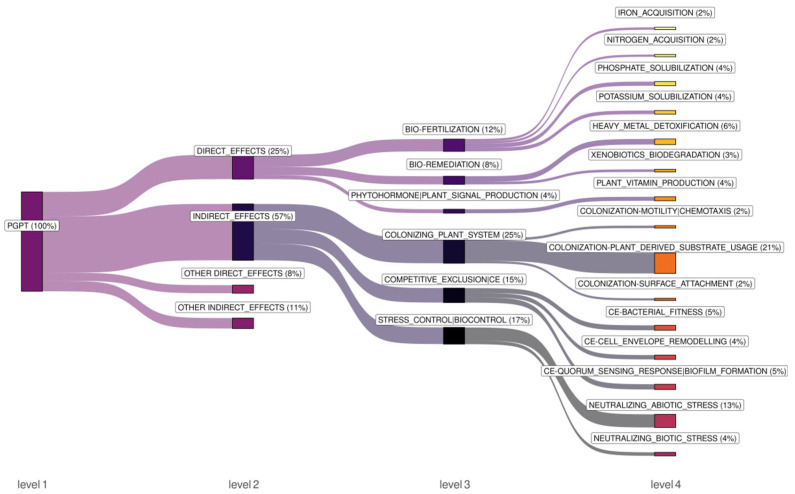
PGPT functional analysis of bacterial endophytic communities in *Picea jezoensis.* The top 15 most abundant categories at level 4 of the PLaBase PGPT ontology are displayed, while the remaining 30 level 4 categories are grouped under “OTHER DIRECT_EFFECTS” or “OTHER INDIRECT_EFFECTS” (Figure S2). Relative abundance of KO copies for each category is shown.

Among the PGPTs, the top 15 most abundant categories at level 4 of the ontology accounted for 25% of the relative abundance of KOs copies associated with direct effects, and 57% associated with indirect effects (Figure 6). Direct effects included bio-fertilization (12%), bio-remediation (8%), and phytohormone/plant signal production (4%). Bio-fertilization encompassed iron acquisition (2%) via siderophores (Figure S3), nitrogen acquisition (2%) primarily through ammonium assimilation, denitrification, urea usage, and atmospheric nitrogen fixation (Figure S4), as well as phosphate (4%) and potassium (4%) solubilization mainly through organic acid metabolism (Figure S5). Bio-remediation involved heavy metal detoxification (6%) predominantly affecting iron, nickel, cobalt, arsenic, copper, and antimony (Figure S6), as well as xenobiotic biodegradation (3%) targeting hydrocarbons, styrene, and benzoate (Figure S7). Phytohormone/plant signal production included plant vitamin production (4%), mainly involving vitamins B9, B5, B12, and B1 (Figure S8).

Indirect effects comprised colonizing plant system (25%), competitive exclusion (15%), and stress control/biocontrol (17%) (Figure 6). The colonizing plant system category included plant-derived substrate usage (21%) involving nucleosides, amino acids, organic acids, carbohydrates, lipids, etc. (Figure S9), surface attachment (2%), and motility/chemotaxis (2%). Competitive exclusion encompassed bacterial fitness (5%), cell envelope remodeling (4%), and quorum-sensing response/biofilm formation (5%) (Figure 6). Stress control/biocontrol involved neutralizing abiotic stress (13%), primarily addressing salinity, oxidative, and osmotic stresses (Figure S10), as well as neutralizing biotic stress (4%) through bactericidal compounds, resistance volatiles, and fungicidal compounds (Figure S11).

Our findings revealed that the structure of the PGPTs varies in different parts of the spruce tree. The most abundant category at level 4 of the ontology was “plant-derived substrate usage” (21%) (Figure 6). Within this category, the endophytic bacteria of branches and needles exhibited an elevated relative abundance of KOs copies for sugar acid, carbohydrate-pentoses, and sugar alcohol utilization, while the microbiome of fresh wood displayed an increased relative abundance of KOs copies for aromatic/phenolic compound and terpene utilization (Figure S9). In the “neutralizing biotic stress” category, both fresh wood and needles microbiomes exhibited an elevated relative abundance of KOs copies for insecticidal compounds (Figure S11). No significant differences in structure of PGPTs were detected between winter and summer and between two spruce specimens.

### 2.4. Diversity and Composition of Endophytic Fungi in Different Tree Parts and Tree Specimens of Picea jezoensis in Winter and Summer

**Figure 7:**
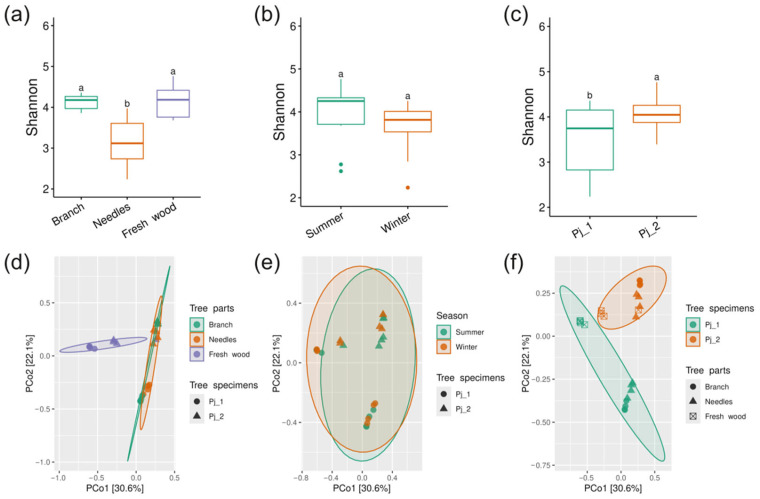
Shannon indexes of endophytic fungal communities in different tree parts (**a**), seasons (**b**), and tree specimens (**c**). PCoA based on Bray–Curtis dissimilarity for endophytic fungal communities in different tree parts (**d**), seasons (**e**), and tree specimens (**f**). Different lowercase letters indicate significant differences among Shannon indexes. Ellipses in PCoA plots show the 95% confidence intervals of a multivariate t-distribution.

Shannon’s diversity index was lower in the needle fungal communities compared to branch and fresh wood fungal communities (*p* < 0.05) (Figure 7a). The Shannon index revealed no significant differences between summer and winter communities (Figure 7b). Shannon’s index indicated lower diversity in Pj_1 plant fungal community compared to Pj_2 (*p* < 0.05) (Figure 7c).

PCoA ordination showed that the fungal communities in branches and needles formed overlapping clusters, with fresh wood communities forming a separate cluster (Figure 7d). The PERMANOVA test revealed significant differences among the communities in different spruce tree parts (R^2^ = 0.37, *p* < 0.01) (Table S8). PCoA indicated substantial overlap between fungal community structures in winter and summer (Figure 7e). PERMANOVA did not confirm significant compositional differences between seasonal fungal communities (Table S8). Fungal communities of tree specimens displayed distinct clustering with slight overlap, as shown by PCoA analysis (Figure 7f). PERMANOVA results confirmed significant differences in fungal community structure between Pj_1 and Pj_2 (R^2^ = 0.22, *p* < 0.01) (Table S8).

**Figure 8:**
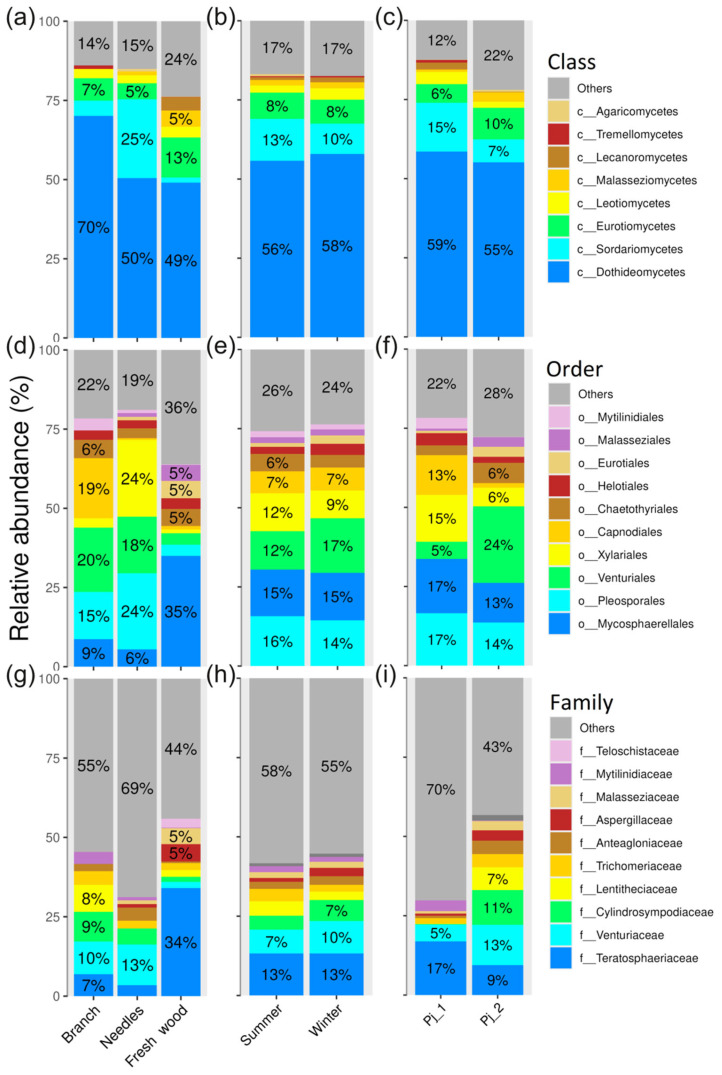
Composition of endophytic fungal communities at class (**a**–**c**), order (**d**–**f**) and family (**g**–**i**) levels in *Picea jezoensis* in different tree parts (**a**,**d**,**g**), seasons (**b**,**e**,**h**), and tree specimens (**c**,**f**,**i**). The top 10 classes, orders, or families are shown. The remaining taxa are grouped into the other category.

The dominant fungal classes associated with *P. jezoensis* were represented by Dothideomycetes, Sordariomycetes, and Eurotiomycetes (Figure 8a–c). The most abundant orders included Mycosphaerellales, Pleosporales, Venturiales, and Xylariales (Figure 8d–f), while the dominant families were Teratosphaeriaceae and Venturiaceae (Figure 8g–i). Notably, a significant proportion of fungal ITS sequences remained unclassified at the family level (Figure 8g–i, Table S9), which may indicate the presence of potentially novel or uncultured fungal taxa within the community.

**Figure 9:**
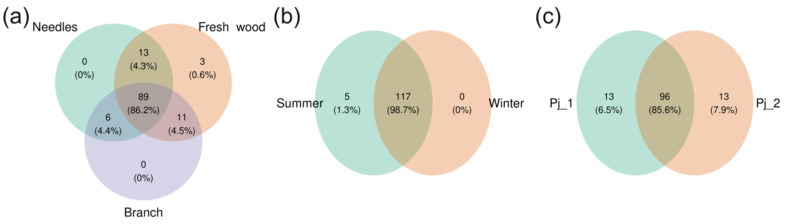
Venn diagrams illustrating shared and unique ASVs in fungal communities of different tree parts (**a**), seasons (**b**), and tree specimens (**c**).

A total of 122 fungal ASVs were found in the endophytic microbiome of *P. jezoensis* (Table S9). Eighty-nine ASVs (86.2%) were common to all analyzed parts of the spruce, and three ASVs were unique to fresh wood (Figure 9a, Table S10). One hundred and seventeen endophytic fungal ASVs (98.7%) were common to two studied seasons (Figure 9b, Table S11). Five unique ASVs were found in the summer (Figure 9b, Table S11). For two tree specimens, 96 ASVs (85.6%) were common (Figure 9c, Table S12). Thirteen unique ASVs were found in Pj_1, and thirteen in Pj_2 (Figure 9c, Table S12).

**Figure 10:**
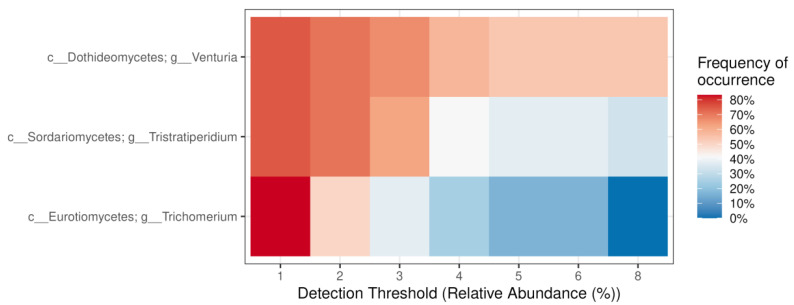
Core endophytic fungal genera in *Picea jezoensis*. The “frequency of occurrence” represents the proportion of samples in which a genus was detected. Genera with relative abundance greater than 1% in at least 50% of the samples are displayed. “c__”—class, “g__”—genus.

Based on relative abundance > 1% in a minimum of 50% samples, the core genera of endophytic fungi in *P. jezoensis* are *Venturia*, *Tristratiperidium*, and *Trichomerium* (Figure 10). Less abundant fungal genera detected in *P. jezoensis* include *Acrodontium*, *Perusta*, *Anteaglonium*, *Lapidomyces*, *Lophium*, *Septonema*, *Malassezia*, *Penicillium*, *Caloplaca*, *Aspergillus*, *Calycina*, *Devriesia*, *Bullanockia*, *Hormonema*, *Capnobotryella*, *Proliferodiscus*, *Paraconiothyrium*, *Arachnopeziza*, *Neosetophoma*, *Myriangium*, *Neocatenulostroma*, *Dothiora*, *Aureobasidium*, *Paradevriesia*, *Monochaetia*, and *Parapyrenochaeta* (Table S9).

**Figure 11:**
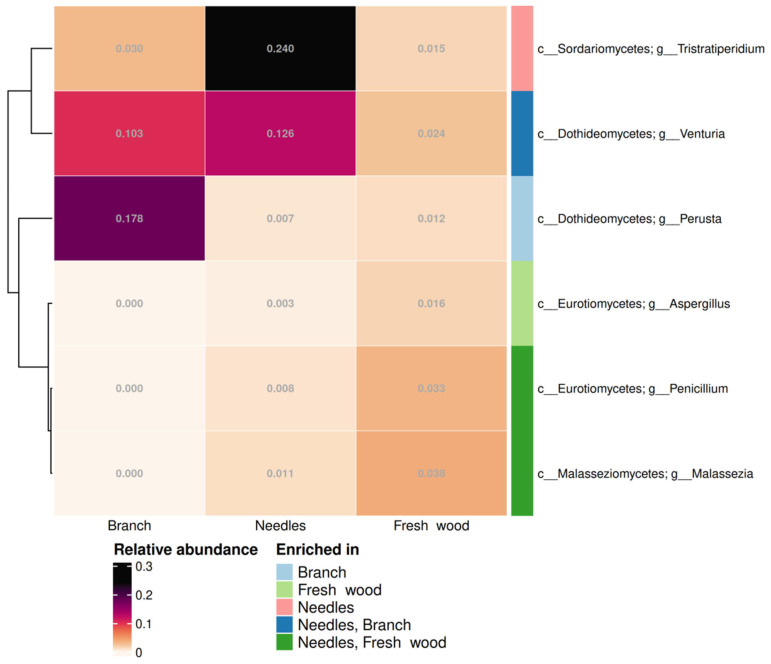
Endophytic fungal genera that show a significant difference in their relative abundance among different tree parts of *Picea jezoensis*. “c__”—class, “g__”—genus. Distinct colors in the “Enriched in” column represent fungi that are significantly enriched in different tree parts of *P. jezoensis* (*p* < 0.05).

According to the differential abundance test, one genus of endophytic bacteria was enriched in the branches, one in the fresh wood, one in the needles, one in the needles and branches, and two in the needles and fresh wood (*p* < 0.05) (Figure 11). Based on relative abundance, the enriched fungal genera in branches are *Perusta* and *Venturia*; in needles, *Tristratiperidium*, *Venturia, Penicillium*, and *Malassezia*; and in fresh wood, *Penicillium*, *Malassezia*, and *Aspergillus* (*p* < 0.05) (Figure 11). In Pj_1 samples, the following genera were dominant: *Acrodontium*, *Lophium*, and *Parapyrenochaeta* (*p* < 0.05). In contrast, Pj_2 samples exhibited enrichment of *Septonema* and *Arachnopeziza* (*p* < 0.05) (Figure S12). Notably, statistical analysis did not reveal any significant variations in fungal genera abundance in winter and summer.

## 3. Discussion

Spruce wood of *P. jezoensis* is widely used in construction, woodworking, and forestry [22]. The *P. jezoensis* bark contains significant amounts of tannin (5–13%) and is used as a tanning agent. The dried shoots contain essential oil (1.5–1.7%), which can be used in perfumery. The bark also contains stilbenes and triterpenoids, substances of great pharmacological value. Because of the extensive use in relatively accessible areas, the Yezo spruce may be threatened with extinction [13].

Despite the ubiquity of endophytes, diversity of endophytes in conifers, and therefore their impact on plant productivity and fitness, has not been well studied. Dark septate root endophytic fungi have been shown to increase growth of Scots pine seedlings under elevated CO_2_ by improving nitrogen use efficiency [23]. Therefore, endophytes may offer opportunities for forestry as growth promoters and for improving the health of woody plants. Currently, biologicals based on endophytic and rhizospheric bacteria have been developed and are widely used for agricultural crops [24]. However, this positive experience has not been applied to forestry, and, to date, no biological products have been developed for forest crops. In order to be able to develop endophyte-based biologicals for the protection of forest trees, it is necessary to have an understanding of their diversity.

In this study, the diversity of endophytic bacteria and fungi of spruce in different aboveground parts (needles, branches, freshly ground wood in the form of sawdust—fresh wood) and in different seasons (winter, summer) in Primorsky Territory was investigated for the first time.

Our study reveals that the needle has low alpha-diversity of both bacterial and fungal communities (Figure 1a and Figure 7a). Yezo spruce needles are characterized by high concentrations of protective substances—terpenes, phenolic compounds, and other metabolites [13,14,15,16]. These substances, while protecting the spruce from herbivores and pathogens, simultaneously inhibit the growth of many microorganisms. The relatively simple internal structure of needles limits the range of conditions in which microorganisms can survive and grow, making them less suitable for microbial colonization. Additionally, the regular shedding of needles prevents microbial communities from having enough time to develop a high level of diversity. In contrast, branches exhibit high alpha-diversity of both bacterial and fungal communities (Figure 1a and Figure 7a). The more stable internal environment, active movement of water and nutrients, accumulation of organic matter, and greater age of branches compared to needles create favorable conditions for the formation and development of diverse microbial communities. Interestingly, fresh wood is favorable for fungi in terms of alpha-diversity, but not for bacteria, when compared to branches (Figure 1a and Figure 7a). Fresh wood is a rich source of complex carbohydrates, such as cellulose and lignin, which can promote the growth of various fungal species that are specialized in breaking down these compounds [25]. It is possible that, while wood is rich in carbohydrates, it may also be relatively poor in other essential nutrients needed for the rapid growth of bacteria. Additionally, fungi may be better adapted to colonizing fresh wood compared to bacteria.

Our research found no significant differences in the alpha-diversity of bacterial and fungal communities on spruce trees between the summer and winter seasons (Figure 1b and Figure 7b). It can be hypothesized that the bacterial and fungal communities within spruce trees may be relatively stable and well-protected from external seasonal fluctuations. However, further research with a larger sample size across all seasons is needed to confirm this hypothesis.

Interestingly, the two trees did not differ in bacterial alpha-diversity, while fungal alpha-diversity was higher in the Pj_2 tree compared to the Pj_1 tree (Figure 1c and Figure 7c). This suggests that, unlike bacteria, fungal communities are more sensitive to individual differences in spruce trees. This could be due to genetic variation between tree specimens, leading to different chemical compositions or physical defenses. Additional research is needed to determine the impact of individual tree differences on fungal communities.

The community of endophytic bacteria in spruce trees primarily differs depending on the tree parts (Figure 1). However, the composition and structure of endophytic bacteria in *P. jezoensis* can remarkably differ from those observed in other coniferous species such as *Pinus arizonica*, *Abies religiosa*, *Pinus contorta var. latifolia, Pinus flexilis, Pinus contorta,* and *Pinus pinaster* [26,27,28,29,30,31]. Thus, the identity of the host species may control the composition of the endophyte bacterial community.

Our metagenomic analysis data demonstrate that the genera *Pseudomonas* and *Methylobacterium-Methylorubrum* exhibit high abundance in *P. jezoensis* trees (Figure 4). In Norway spruce seeds, non-pathogenic members of the genus *Pseudomonas* have been found to have growth-stimulating activity in experiments [32]. *Methylobacterium* spp. are the dominant representatives of the endophytic microbiota of Scots pine (*Pinus sylvestris*) buds [33], whose symbiosis positively influences the growth and development of the host plant. It suggests that endophytic bacteria from these genera may also positively impact the growth and development of spruce. Thus, some species of endophytic bacteria may be useful for stimulating the growth of coniferous trees or inhibiting pathogenic microorganisms of woody plants, but this assumption requires further research.

The most abundant PLaBAse PGPT level 3 category within direct effects is bio-fertilization, which may indicate a significant effect of the endophytic bacteria in nutrient acquisition (iron, nitrogen, phosphate, and potassium) for the plant (Figure 6). The largest proportion of KO copies among PLaBAse PGPT level 3 categories was associated with the indirect effect “colonizing plant system” (Figure 6). This suggests that the bacteria’s ability to establish themselves within the plant and utilize plant-derived resources is a crucial aspect of their plant growth-promoting capabilities. Notably, our results demonstrate that the structure of KO copies responsible for the usage of plant-derived substrates exhibits significant variation across different parts of the spruce tree. Within this category, the endophytic bacteria of branches and needles exhibited an elevated relative abundance of KO copies for sugar acid, carbohydrate-pentoses, and sugar alcohol utilization (Figure S9). The needles of the spruce are photosynthetic, and the branches are involved in the export of carbohydrates [34], so these parts of the spruce would be rich in these types of carbon sources. The endophytic bacteria of fresh wood displayed an increased abundance of KO copies for aromatic/phenolic compound and terpene utilization (Figure S9). It is known that spruce fresh wood is a rich source of stilbenes and terpenes [13]. This suggests that the endophytic communities adapt their functional capabilities based on the specific microenvironment and resource availability within different plant parts.

Interestingly, “neutralizing abiotic stress” accounts for 13% of KO copies, which may indicate the bacteria’s ability to mitigate salinity, oxidative, and osmotic stresses for the plant, which is highly beneficial for survival in challenging environments (Figure S10). “Neutralizing biotic stress” accounts for 4% of KO copies, indicating potential direct antagonistic effects against plant pathogens (Figure S11). Both fresh wood and needles microbiomes exhibited an elevated relative abundance of KO copies for insecticidal compounds, suggesting a shared protective role against insects in these tissues (Figure S11).

While in silico functional predictions remain valuable tools, they should be interpreted with caution. PICRUSt2 still provides limited insights into bacterial communities based on rRNA gene analysis. Unfortunately, PICRUSt2 cannot be applied to assess the functional potential of fungal communities. Metatranscriptomics currently represents the most reliable method for elucidating functional profiles within microbial ecosystems, providing a deeper understanding of plant–microbe interactions [35,36,37,38]. Furthermore, the application of metatranscriptomic approaches can facilitate the identification of the most promising biocontrol agents for fungal diseases of woody plants [38]. As a more advanced approach, spatial metatranscriptomics facilitates the investigation of both inter- and intrakingdom spatial interactions within microbial communities and provides insights into the host response to microbial hotspots [39]. However, the implementation of metatranscriptomics and spatial metatranscriptomics is significantly limited by their complexity and high cost.

The composition of endophytic fungi in Yezo spruce was different from the previously analyzed endophytic fungal communities of other conifers such as *Abies koreana Abies nephrolepis*, *Thuja koraiensis*, *Pinus contorta*, and *Pinus flexilis* [35,40,41,42]. Thus, the host species’ identity could regulate the composition of the fungal endophyte community.

According to our data, *Venturia*, *Tristratiperidium*, and *Trichomerium* were predominant in *P. jezoensis* (Figure 10). The genus *Venturia* primarily comprises phytopathogenic fungi known to cause substantial economic damage to fruit crops worldwide, particularly as causative agents of scab diseases [43]. To date, no diseases of spruce trees caused by *Venturia* species have been reported. Interestingly, *Venturia* was detected in the needles of *Picea glauca*, where it exhibited a dominant presence alongside the genera *Cladosporium* and *Tryblidiopsis* [44]. *Tristratiperidium* is a genus of fungi represented by the single species, *T.* *microsporum* [45]. The genus *Tristratiperidium* was previously detected in the phyllosphere of healthy vegetative buds of *Picea abies* [46]. Species of *Trichomerium* are classified as sooty molds [47]. Their main food source is the sugary secretions produced by sap-sucking insects, such as aphids, mealybugs, and scabies, as well as natural plant excretions. *Trichomerium* was previously detected on healthy needles of *P. abies* and *Abies alba* at relatively high abundance [48]. Given that our sampling targeted visually healthy tissues, these genera may function as symbiotrophs within the Yezo spruce, a hypothesis requiring further investigation.

Endophytic fungal communities in *P. jezoensis* differ depending on tree parts and specimens (Figure 7). Notably, *Penicillium* exhibited an increased proportion of fresh wood and needles (Figure 11). Additionally, in the fresh wood of *P. jezoensis*, we observed an elevated abundance of *Aspergillus* spp. compared to other tree parts (Figure 11). It is noteworthy that *Penicillium* and *Aspergillus* genera, whose species are often soil saprotrophs, exhibit high abundance in fresh wood. It is possible that species of these genera function as opportunistic endophytes in Yezo spruce fresh wood, and their high abundance may be attributed to the fact that the wood cambium is a constantly growing tissue. During its growth process, dead tissue is formed, which can serve as an additional food source for these microorganisms. It is known that some species of these very diverse genera inhibit the growth of disease-causing fungi. Previous studies demonstrated that *Penicillium* fungi from *Picea glehnii* seeds protect seedlings from the pathogenic *Pythium vexans* [49]. The earlier investigations have demonstrated the biocontrol potential of *A. terreus* against pathogenic fungi, such as *Rigidoporus microporus* and *Corynespora cassiicola*, in woody plants, particularly in the rubber tree [50]. Furthermore, endophytic *A. terreus* has been shown to possess insecticidal properties [51]. This suggests that the fungal endophytic community in Yezo spruce may contribute to plant defense against pathogens.

However, there is a need to isolate the strains of bacterial and fungal endophytes from the spruce tissues in order to analyze their biological and metabolic properties. This approach is crucial for understanding the role of endophytes in ecosystems and agrobiotechnology. In the future, we plan to conduct analysis using modern methods of cultivation and molecular genetic analysis, which will allow us to more accurately identify endophytes in Yezo spruces that can be useful in agricultural applications.

## 4. Materials and Methods

### 4.1. Site Description

**Figure 12:**
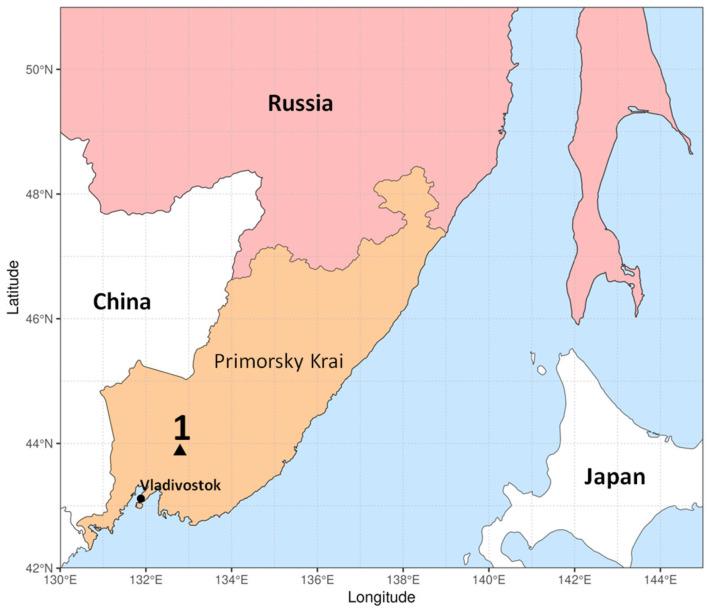
The map of sampling area. 1—spruce material collection site Ivanovka.

Samples of visually healthy *P. jezoensis* trees (Pj-1 and Pj-2) were collected in the forest near the village of Ivanovka, in the center of Primorsky Territory, Russian Far East (Figure 12). The sampled trees were approximately 15 m in height and 20–25 years old.

### 4.2. Sampling

Samples of needles, branches, and freshly ground wood in the form of sawdust (- fresh wood) were collected from two trees in February and July 2023. The characteristics of weather in the region are given in Table 1. The samples were collected into sterile plastic bags and were delivered to the lab within 1–2 days. A total of 24 biological replicates (2 of each tree part from each tree specimen and in each season) were sampled and analyzed (Tables S1 and S2).

### 4.3. DNA Extraction, Library Preparation, and Illumina MiSeq Sequencing

DNA for NGS was extracted from needles, branches, and wood chips of *P. jezoensis* trees using the CTAB spin technique as described previously [52,53,54]. For advanced sequencing using the Illumina technology, the DNA samples were sent to Syntol in Moscow, Russia. DNA quality and quantity were assessed using the Nanodrop-1000 (Nanodrop, Wilmington, DE, USA) and Quantus fluorometer (Promega, Madison, WI, USA), respectively. Sequenced bacterial and fungal libraries were constructed according to a detailed protocol provided by the manufacturer of the sequencing platform “16S Metagenomic Sequencing Library Preparation” (Part # 15,044,223 Rev. B; Illumina, San Diego, CA, USA). This protocol can also be used for the preparation of a fungal library. Bacterial 16S rRNA regions and fungal ITS1 rDNA regions were amplified from the samples using the primers described earlier [52,53]. Amplicons were indexed using the Nextera^®^ XT Index Kit. The library pool was sequenced on Illumina MiSeq platform (2 × 250 paired-end) using MiSeq Reagent Kit v2 with 500-cycle paired-end reads.

Sequences of the spruce bacterial and fungal endophytes have been deposited at NCBI under accession number PRJNA1210692.

### 4.4. Bioinformatics Data Analysis

The data used for the bioinformatic analysis can be found in Tables S1 and S2. Custom scripts written in R and Bash were used to process the data (https://github.com/niknit96/Aleynova_et.al.2025 (accessed 18 June 2025)). For 16S data, the initial reads were preprocessed using QIIME 2 (version 2024.10.1) [55] and DADA2 (version 2024.10.0) (with options: denoise-paired, --p-trim-left-f 21, --p-trunc-len-f 160, --p-trim-left-r 21, --p-trunc-len-r 142) [56], which included the elimination of primers and chimeric sequences, followed by the merging of paired-end reads. For ITS data, we removed primers using cutadapt and utilized only the forward read in the DADA2 step (with options: denoise-single, --p-trunc-len-f 187) due to the poor quality of the reverse read. Taxonomic identification was performed using the QIIME 2 Scikit-learn algorithm in conjunction with the SILVA 138 pre-trained classifier for 16S sequences [57], and the UNITE pre-trained classifier for ITS sequences [58].

The packages qiime2R (version 0.99.6) [59], phyloseq (version 1.50.0) [60], microeco (version 1.16.0) [61], microViz (version 0.12.7) [62], microbiome (version 1.23.1) [63], and tidyverse (version 2.0.0) [64] were used for the initial filtering and data preparation. Sequences corresponding to mitochondria, chloroplasts, non-bacteria, and non-fungi were excluded from the data set. Samples were rarefied to an even depth (10,000 reads per sample), and amplicon sequence variants (ASVs) were filtered based on a relative abundance threshold of >0.1% and minimum occurrence in 5 samples of spruce.

Shannon index and Bray–Curtis dissimilarity were calculated using the microeco R package for the characterization of alpha- and beta-diversity, respectively, of bacterial and fungal endophytic communities. The Shannon alpha diversity metric was calculated on normalized samples without filtration of ASVs. An analysis of variance (ANOVA) was used to compare alpha diversity among groups (different tree parts, seasons, or tree specimens of *P. jesoensis*). Bray–Curtis dissimilarity data were used to perform the principal coordinates analysis (PCoA) in the microeco R package. Beta diversity data were statistically validated using a permutation-based analysis of variance (PERMANOVA) with 999 permutations. The ANCOM-BC2 method was used to identify taxa that were differentially abundant among groups [65].

PICRUSt2 (version 2.6.1) [66] and the PLaBAse PGPT ontology [67] were used for functional analysis of endophytic bacterial communities. PICRUSt2 predicts the KEGG orthologs (KOs) copies in a microbial community based on 16S rRNA gene data using known genomes of related organisms. KO group genes and proteins from diverse species that are predicted to carry out the same specific molecular function within the context of KEGG metabolic pathways and molecular networks [68]. PLaBAse PGPT ontology is a literature- and OMICs-curated, comprehensive, and hierarchical gene ontology of bacterial traits associated with plant growth-promotion. The PICRUSt2-predicted KOs were mapped to the PLaBAse PGPT ontology using tidyverse tools. ANCOM-BC2 was used to identify potential PGPT functional capabilities of bacterial communities that were differentially abundant among groups.

For statistical analyses involving ANOVA, PERMANOVA, and ANCOM-BC2, we applied Benjamini–Hochberg corrections to account for multiple testing. Statistical significance was determined at a threshold of *p* < 0.05 following the correction.

Results were visualized using the microeco, microbiome, ggsankey (version 0.0.99999) [69] and ggplot2 (version 3.5.2) [64] packages. Microeco and ggplot2 were employed for Figure 1, Figure 2, Figure 3, Figure 7, Figure 8 and Figure 9; microbiome was used for Figure 4, Figure 5, Figure 10, Figure 11, Figures S1 and S3–S12; ggsankey was applied for Figure 6 and Figure S2.

## 5. Conclusions

The presented study was the first to investigate the diversity of endophytic bacteria and fungi in Yezo spruce in different above-ground parts (needles, branches, and fresh wood) and during different seasons (winter, summer) in Primorsky Territory. Our findings indicate that the composition of endophytic bacterial and fungal communities in *P. jezoensis* spruce is specific and primarily determined by tree parts (for both bacteria and fungi) and by different tree specimens (for fungi). This study provides valuable insights into the intricate functional relationships between bacterial endophytes and their host plants. The high prevalence and diversity of PGPTs in *P. jezoensis* endophytic bacteria suggest their significant potential as bio-inoculants for sustainable forestry and agriculture. They could be used to enhance nutrient uptake, improve stress tolerance, and provide disease resistance, reducing the reliance on synthetic fertilizers and pesticides. Some endophytic fungi may exhibit antagonistic activity against pathogenic fungal species. Since approaches based on short-read amplicon sequencing have limited resolution for genus- or species-level identification and functional analysis, future research could focus on the application of novel sequencing technologies, such as long-read sequencing and metatranscriptomics, to enhance capabilities for detailed taxonomic resolution and comprehensive functional characterization of microbial communities. Additionally, research efforts should include the isolation and characterization of specific endophytic strains with potent PGPTs, followed by validation of their effects through controlled laboratory experiments and field trials, as well as exploration of the molecular mechanisms underlying these beneficial interactions.

## Figures and Tables

**Table 1:** Average temperature and precipitation in Ivanovka region, Primorsky Territory.

Month	Average t, °C	Precipitation, mm
February	−8	24
July	25	100

## Data Availability

The data presented in this study are available within the article and Supplementary Material.

## Supplementary Materials

The following supporting information can be downloaded at: https://www.mdpi.com/article/10.3390/plants14162534/s1. Table S1. 16S data samples used in analysis; Table S2. ITS data samples used in analysis; Table S3. PERMANOVA results (16S data); Table S4. Bacterial ASVs and their relative abundance in different tree parts, seasons, and tree specimens of *Picea jezoensis*; Table S5. Overlapping ASVs in the 16S metagenomic data of different tree parts of *Picea jezoensis*; Table S6. Overlapping ASVs in the 16S metagenomic data of *Picea jezoensis* according to season of sampling; Table S7. Overlapping ASVs in the 16S metagenomic data in different tree specimens of *Picea jezoensis*; Figure S1. Endophytic bacterial genera that show a significant difference in their relative abundance of sequences between different seasons of *Picea jezoensis*; Figure S2. Thirty low-abundant PLaBase PGPT level 4 categories in functional analysis of bacterial endophytic communities in *Picea jezoensis*; Figure S3. Composition of KOs copies associated with “iron acquisition” in bacterial endophytic community of different tree parts of *Picea jezoensis*; Figure S4. Composition of KOs copies associated with “nitrogen acquisition” in bacterial endophytic community of different tree parts of *Picea jezoensis*; Figure S5. Composition of KOs copies associated with “phosphate solubilization” and “potassium solubilization” in bacterial endophytic community of different tree parts of *Picea jezoensis*; Figure S6. Composition of KOs copies associated with “heavy metal detoxification” in bacterial endophytic community of different tree parts of *Picea jezoensis*; Figure S7. Composition of KOs copies associated with “xenobiotics biodegradation” in bacterial endophytic community of different tree parts of *Picea jezoensis*; Figure S8. Composition of KOs copies associated with “plant vitamin production” in bacterial endophytic community of different tree parts of *Picea jezoensis*; Figure S9. Composition of KOs copies associated with “plant-derived substrate usage” in bacterial endophytic community of different tree parts of *Picea jezoensis*; Figure S10. Composition of KOs copies associated with “neutralizing abiotic stress” in bacterial endophytic community of different tree parts of *Picea jezoensis*; Figure S11. Composition of KOs copies associated with “neutralizing biotic stress” in bacterial endophytic community of different tree parts of *Picea jezoensis*; Table S8. PERMANOVA results (ITS data); Table S9. Fungal ASVs and their relative abundance in different tree parts, seasons, and tree specimens of *Picea jezoensis*; Table S10. Overlapping ASVs in the ITS metagenomic data of different tree parts of *Picea jezoensis*; Table S11. Overlapping ASVs in the ITS metagenomic data of *Picea jezoensis* according to season of sampling; Table S12. Overlapping ASVs in the ITS metagenomic data in different tree specimens of *Picea jezoensis*; Figure S12. Endophytic fungal genera that show a significant difference in their relative abundance of sequences between different tree specimens of *Picea jezoensis*.
